# Mechanical Circulatory Support on Coronary Artery Bypass Grafting for Advanced Ischemic Cardiomyopathy: State of the Art

**DOI:** 10.3390/medicina62040638

**Published:** 2026-03-27

**Authors:** Erik J. Orozco-Hernandez, James E. Davies, Sasha Anne Still, Kyle W. Eudailey, Venkateswaran Rajamiyer, Panos N. Vardas, Clifton T. Lewis, William Holman

**Affiliations:** Division of Cardiothoracic Surgery, Department of Cardiac Surgery, University of Alabama at Birmingham, Birmingham, AL 35294, USA

**Keywords:** ischemic cardiomyopathy, left ventricular dysfunction, myocardial viability, hibernating myocardium, coronary artery bypass grafting, mechanical circulatory support

## Abstract

Ischemic cardiomyopathy is defined as coronary artery disease accompanied by left ventricular dysfunction with an ejection fraction equal to or less than 40%. The substrate of ischemic cardiomyopathy is heterogeneous, characterized by the coexistence of normal, stunned, hibernating, and scarred myocardium within the same myocardial region. Altogether, these components may represent different phases of a single pathological process. It is well-established that the assessment of isolated myocardial viability and ischemia alone has failed to reliably guide the indication for coronary artery bypass grafting (CABG). CABG in patients with low ejection fraction carries a significant risk of perioperative mortality and morbidity, largely related to the development of postcardiotomy shock. Preoperative optimization with pharmacologic or mechanical circulatory support (MCS) is often essential; the decision requires integrating multiple complex factors, including clinical presentation, response to optimization therapy, myocardial viability, the presence of hibernating or scarred myocardium, left ventricular end-systolic volume index, coronary angiography findings, hemodynamic assessment, and the Pulmonary Arterial Pressure Index score. A preoperative evaluation that incorporates anatomical, morphological, functional, and hemodynamic domains enables more precise selection and timing of MCS. Preemptive left ventricular unloading mitigates the physiological impact of cardiopulmonary bypass, preserves end-organ perfusion, and reduces the need for high-dose vasopressors. However, the risk–benefit ratio remains uncertain and may be associated with serious complications. Careful judgment regarding the indications for MCS has the potential to enhance the safety of CABG in high-risk patients, but robust, long-term, prospective studies are needed to determine its true impact on clinical outcomes. In this review, we will examine the indications and criteria for the use of MCS in patients with advanced ischemic cardiomyopathy, as well as the various devices available for preoperative or intraoperative support, including technical considerations, advantages and disadvantages, and associated complications.

## 1. Introduction

Coronary artery disease (CAD) is the leading cause of heart failure worldwide. Advanced ischemic cardiomyopathy represents one of the most challenging phenotypes within the spectrum of chronic coronary disease, where progressive ventricular remodeling, microvascular dysfunction, and recurrent ischemic injury converge to produce severe systolic impairment. In this population, CABG remains the only revascularization strategy with a demonstrated long-term survival benefit, a finding supported by U.S., Asian, and European revascularization and heart failure guidelines [1,2,3]. Yet the procedure is frequently undertaken in a hemodynamic environment characterized by low cardiac output, limited contractile reserve, and heightened perioperative risk. As surgical candidates become older and more comorbid, the physiological margin for error narrows, prompting renewed interest in strategies that can stabilize circulation and optimize myocardial perfusion during the perioperative period.

Within this context, the intent of mechanical circulatory support (MCS) is not confined to a single purpose: it may serve a therapeutic role—stabilizing preoperative heart failure or ischemia to allow patients to reach surgery in a more favorable physiological state—or a prophylactic role, when deployed anticipatorily in patients at high risk of intraoperative or postoperative hemodynamic collapse.

MCS has emerged as a pivotal adjunct in this setting, offering the ability to unload the ventricle, augment systemic perfusion, and create a more favorable substrate for surgical revascularization. Devices ranging from intra-aortic balloon pumps to contemporary micro-axial pumps and extracorporeal membrane oxygenation have expanded the therapeutic armamentarium, yet their optimal timing, selection, and integration into CABG workflows remain subjects of active debate. Evidence suggests that proactive, planned support may confer advantages over rescue implantation, but the heterogeneity of available data and the rapid evolution of technology demand a critical and updated synthesis.

This state-of-the-art review examines the current role of mechanical circulatory support in CABG for advanced ischemic cardiomyopathy, integrating pathophysiological principles, device-specific considerations, and contemporary clinical outcomes. By contextualizing emerging evidence within the broader landscape of surgical revascularization, the review aims to clarify where MCS meaningfully alters risk, where uncertainties persist, and how evolving strategies may reshape the management of this high-risk population.

## 2. Materials and Methods

This narrative, evidence-based review aimed to synthesize the available information on the use of mechanical circulatory support (MCS) in patients with advanced ischemic cardiomyopathy undergoing coronary artery bypass grafting (CABG). A structured methodology was followed to ensure a comprehensive search and methodological rigor in the selection and analysis of the literature.

### 2.1. Search Strategy

A broad search was conducted in the major biomedical databases: PubMed/MEDLINE, Embase, Scopus, Web of Science, Cochrane Library, and Google Scholar. The search period extended from January 2000 to January 2026. MeSH terms and keywords related to ischemic cardiomyopathy, left ventricular dysfunction, myocardial viability, hibernating myocardium, coronary artery bypass grafting, and mechanical circulatory support were used. Terms were combined with Boolean operators (AND, OR). Filters were applied for language (English and Spanish) and adult populations.

### 2.2. Inclusion and Exclusion Criteria

Studies were included if they addressed: adult patients with advanced ischemic cardiomyopathy undergoing CABG; use of any MCS modality in the preoperative, intraoperative, or postoperative period; study designs such as clinical trials, observational studies, registries, case series, prior reviews, and consensus documents; and clinically relevant outcomes, including mortality, perioperative complications, hemodynamic support, ventricular recovery, and selection criteria. Articles were excluded if they were duplicates, contained insufficient data, involved non-comparable populations, or did not focus on CABG or MCS.

The search identified 36 records across all databases. After removal of duplicates (none identified in the final curated list), all 36 full-text articles were assessed for eligibility. A total of 36 studies met the inclusion criteria and were incorporated into the narrative synthesis. These comprised 13 MCS-focused clinical studies, 5 studies on CABG in left ventricular dysfunction, 10 investigations on myocardial viability and ischemic burden, 4 major clinical practice guidelines or consensus documents, 5 device-specific reports, and 1 study addressing social determinants of MCS use. No additional studies were excluded after full-text review.

### 2.3. Ethical Considerations

As this study is a review of previously published literature, approval from an ethics committee was not required.

### 2.4. Thematic Development and Analysis

Left ventricular ejection fraction (LVEF) and heart failure status are the most consistently reported preoperative heart function metrics associated with the need for MCS during CABG [4]. CABG in patients with low ejection fraction carries a significant risk of perioperative mortality and morbidity related to the development of postcardiotomy shock. Preoperative optimization with pharmacological or MCS is beneficial. Multiple series and analyses have found a postoperative mortality rate of between 6.5 and 7.5% [5,6,7].

Multidisciplinary assessment and preoperative planning are essential for optimizing patient selection and timing of MCS in CABG. It is important to understand the heterogeneous substrate of ischemic cardiomyopathy. There is usually coexistence between normal, stunned, hibernating, and scar in the same myocardial region. There is often an element of overlapping between two or more of these states [8].

The STICH trial was a randomized multicenter non-blinded controlled trial that compared medical therapy versus CABG in patients with coronary disease and left ventricular dysfunction [9]. Among the conclusions obtained in that study, the following stand out:

1. Patients assigned to CABG had lower mortality rates and hospitalizations for cardiovascular disease (the difference was borderline *p* 0.05); however, there was no difference between medical therapy and surgery with respect to mortality from any cause.
2. CABG was related to an early risk of mortality. The older the patient, the greater the likelihood of postoperative mortality due to non-cardiovascular causes.
3. There was no benefit of CABG in patients without coronary artery disease in the LAD and/or class III/IV angina.
4. The study was not blinded, and the difference between the two groups, regarding mortality for any cause, may be due to a limited follow-up of the patients.

A STICH viability sub-study reported the effects of myocardial viability (evaluated by DES and SPECT) in 5.1 years of follow-up. Patients with viable myocardium (with or without CABG) were more likely to survive in the univariate analysis; however, this benefit was not demonstrated in the multivariate analysis [10]. It is mandatory to highlight important facts of this study: (a) only half of the STICH study underwent viability studies; and (b) MRI or PET was not used to determine myocardial viability. There is no interaction between the effect of CABG and the presence or absence of viability; the fact of having myocardial viability does not adequately identify which patients would benefit more from surgical revascularization. This sub-study specifically studied patients with myocardial ischemia during stress testing. No benefit of CABG was demonstrated versus medical therapy, based only on the presence or absence of ischemia [11].

The PARR-2 randomized trial demonstrated no statistically significant differences in mortality or major adverse cardiac events between the fluorine-18 fluorodeoxyglucose (FDG) PET-guided treatment and standard care after a 1-year or 5-year follow-up period [7]. However, both the Ottawa-FIVE sub-study of the PARR-2 trial and a post hoc analysis of the PET-guided treatment alone showed a more pronounced benefit of revascularization in patients with extensive hibernation [12,13].

### 2.5. Advanced Ischemic Cardiomyopathy: Imaging Modalities and CABG

As previously mentioned, there is typically a combined coexistence of normal, stunned, hibernating, and scarred myocardium within the same myocardial region. Although the various myocardial states affected by coronary artery disease often coexist along a subtle continuum, it remains essential to understand—objectively and in detail—the capabilities and limitations of the imaging modalities available for evaluating these patients [14,15] (Table 1).

In alignment with this concept, it has been noted that identifying patients with severe LV dysfunction who may benefit from revascularization requires assessing not only hibernation but also the presence and extent of scar tissue, using FDG PET and MRI with late gadolinium enhancement (LGE) (Table 1). Song et al. examined the impact of hibernating myocardium and scar burden using FDG PET and LGE CMR on the survival benefit associated with CABG versus medical therapy alone in patients with severe LV dysfunction. In their study population, CABG was generally associated with a reduced risk of all-cause mortality and a composite of secondary endpoints that included cardiovascular death, cardiovascular hospitalization, heart transplantation, repeat revascularization, ICD implantation, and non-fatal stroke. Notably, patients with extensive hibernating myocardium (>10%) and limited myocardial scar (<26%) derived the greatest benefit from CABG, whereas in patients with extensive scar (>26%) and extensive hibernating myocardium (>10%), hospitalization rates for cardiovascular causes were comparable between CABG and medical therapy [16].

In patients with severely reduced LVEF being evaluated for CABG, the choice of imaging modality depends on the clinical priority [14,15]:

- When the priority is to avoid missing viable myocardium (younger patients, acceptable surgical risk, high potential for recovery), modalities with the highest sensitivity—FDG-PET and CMR-LGE—are preferred. These techniques minimize false negatives and help identify hibernating myocardium that may recover after revascularization.
- When the priority is to avoid grafting non-viable territories (extreme surgical risk, frailty, major comorbidities), modalities with higher specificity—dobutamine stress echocardiography, dobutamine CMR, and Tc-99m SPECT—are more appropriate. These reduce false positives and help prevent unnecessary bypasses to scarred myocardium.
- When surgical planning requires detailed mapping of scar and ischemia, particularly in multivessel disease with diffuse dysfunction, CMR with LGE (±stress perfusion) provides the most comprehensive assessment. It delineates transmural, subendocardial, and patchy fibrosis and identifies territories with inducible ischemia that may benefit from grafting.
- When microvascular dysfunction or balanced ischemia is suspected, PET perfusion with absolute flow quantification offers unique insight that can refine decisions about the extent and expected benefit of CABG.

This framework supports individualized decision-making in high-risk patients, helping determine whether complete revascularization, limited grafting, or alternative strategies are most appropriate (Table 2).

The 2021 American College of Cardiology/American Heart Association/Society for Cardiovascular Angiography and Interventions (ACC/AHA/SCAI) guideline for coronary revascularization assigned a class 1 recommendation, level of evidence B-R for CABG in patients with severe left ventricle (LV) dysfunction (ejection fraction < 35%) [1]. The 2025 Japanese Circulation Society and the Japanese Heart Failure Society Joint Working guideline for coronary revascularization assigned a class IIa recommendation, level of evidence B-R for CABG in patients with severe left ventricle (LV) dysfunction (ejection fraction < 35%) [3].

However, although evaluating myocardial viability and ischemia is necessary, it is not sufficiently comprehensive to determine the indication for CABG with absolute certainty or precision in ischemic cardiomyopathy. Therefore, the analytical approach has turned towards the evaluation of anatomical and hemodynamic variables. Panza et al. studied the following factors in the STICH population: extent of coronary heart disease (three vessels), EF ≤ 27%, and LVESV index ≥ 79 mL/m^2^. Their conclusions led them to recommend surgical revascularization in patients who had two or more previously cited criteria [17].

### 2.6. Advanced Ischemic Cardiomyopathy and CABG: Hemodynamic Evaluation

Some authors recommended that patients with severe ischemic cardiomyopathy should undergo right heart catheterization to identify the degree of cardiogenic shock [cardiac index (CI) < 2.2 L/min/m^2^], the degree of LV decompensation [pulmonary capillary wedge pressure (PCWP) > 20 mmHg] and the degree of right ventricular (RV) dysfunction [Pulmonary Artery Pulsatility Index (PAPi) < 2]. CABG can be performed without delay if there are no signs of cardiogenic shock. If signs of shock are unresponsive to medical treatment alone, the patient may be a candidate to receive mechanical support either preoperatively or intraoperatively [18].

The Pulmonary Artery Pulsatility Index (PAPi) has emerged as one of the most reliable hemodynamic markers of right ventricular (RV) performance in patients with advanced ischemic cardiomyopathy undergoing CABG. A PAPi < 1.5 identifies patients with severe RV dysfunction who are at markedly increased risk of perioperative RV failure, difficulty separating from cardiopulmonary bypass, and need for temporary mechanical circulatory support. Values between 1.5 and 2.0 indicate moderate–severe dysfunction and warrant aggressive preoperative optimization with diuresis, inotropes, and pulmonary vasodilators. In contrast, PAPi values > 2.0–3.0 reflect relatively preserved RV pulsatility and are associated with acceptable operative risk. Because PAPi integrates preload, afterload, and RV contractile reserve, it provides a practical and prognostically meaningful tool for refining surgical candidacy, anticipating perioperative support needs, and tailoring revascularization strategies in patients with severely reduced LVEF [19,20].

Elevated pulmonary artery pressures are consistently identified as a key factor in the decision to use mechanical circulatory support in CABG and related procedures. Monitoring and interpreting pulmonary artery pressure measurements are essential for identifying patients at risk of hemodynamic compromise and for optimizing the timing and type of mechanical support during and after CABG [21].

Right ventricular dysfunction (RVD) is increasingly recognized as a major determinant of perioperative morbidity and mortality in patients undergoing coronary artery bypass grafting (CABG) for advanced ischemic cardiomyopathy. Data from the STICH cohort demonstrate that baseline RVD is independently associated with higher long-term mortality, and that the survival benefit of CABG diminishes progressively as RV dysfunction worsens [9]. These findings underscore the importance of preoperative RV assessment and, in selected cases, preoperative RV support to optimize surgical candidacy.

Initial management focuses on reducing RV preload (diuresis), lowering RV afterload (pulmonary vasodilators such as inhaled nitric oxide or prostacyclin), and augmenting contractility (dobutamine or milrinone). Correction of hypoxia, hypercapnia, and arrhythmias is essential. Although these measures are first-line, they may be insufficient in patients with severe RVD or biventricular failure.

**Figure 1:**
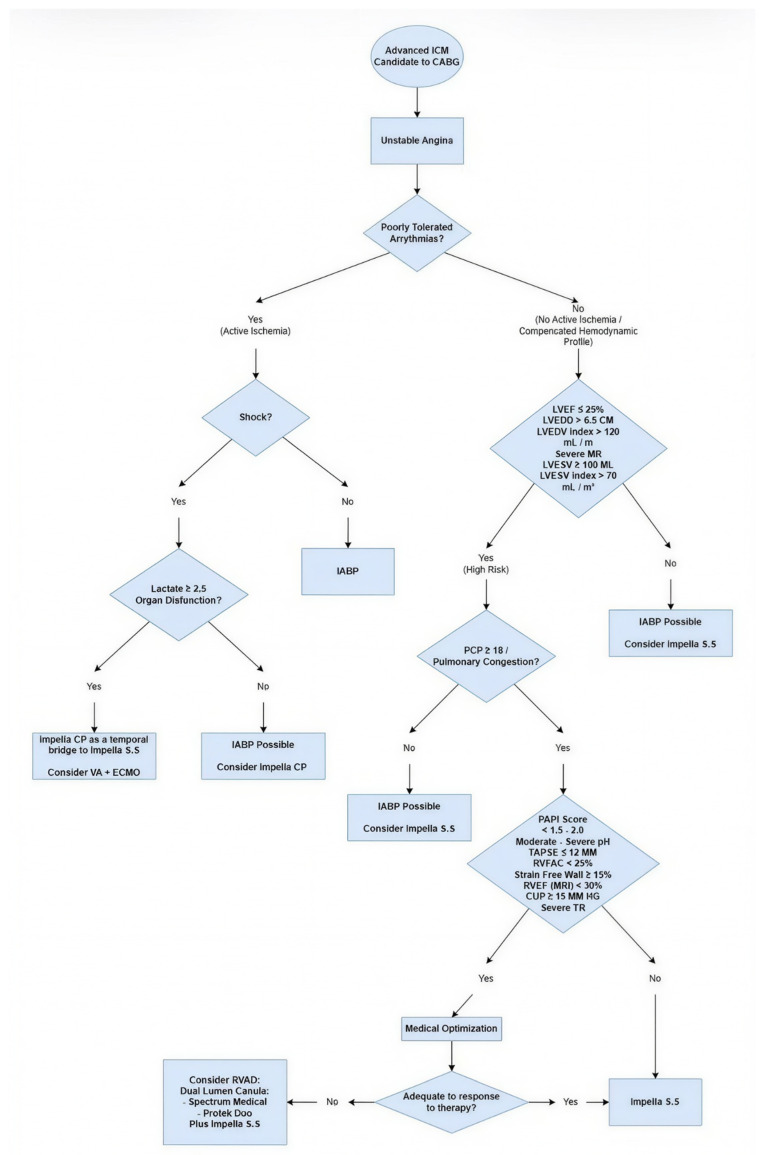
Decision algorithm for mechanical circulatory support in patients with advanced ischemic cardiomyopathy undergoing CABG.

Mechanical RV support may be considered in patients with severe RVD, elevated pulmonary pressures, or hemodynamic instability despite optimal medical therapy [19,20] (Figure 1).

### 2.7. Mcs in Advanced Ischemic Cardiomyopathy in Candidates for CABG

On the other hand, Singh et al. does not evaluate the response to medical treatment, these authors recommended direct pre op MCS (Impella, IABP or ECMO) for patients with cardiogenic shock, including consideration of prophylactic Impella (Abiomed, Danvers, MA, USA) in patients without cardiogenic shock but with very low EF (less than 25%) [4]. This decision represents a complex decision; veno-arterial (VA) extracorporeal membrane oxygenation (ECMO) following cardiac surgery shows an overall survival of between 25% and 42% [22].

Recent analyses of large cardiac surgery databases revealed evolving patterns in the use of mechanical circulatory support (MCS) in patients undergoing coronary artery bypass grafting (CABG), particularly those with acute myocardial infarction complicated by cardiogenic shock. There has been a notable decline in operative mortality for these patients, decreasing from 24.2% in 2011 to 19.0% in 2022, while the annual volume of such procedures has remained stable [23].

Singh et al. [4] found a better survival after CABG with early Impella implantation. Note that all the patients had ischemic cardiomyopathy and post-cardiotomy low cardiac output. A simplistic take on this finding is that axial flow pumps should be used more liberally to diminish post CABG mortality.

There is no information in this small sample about viability and ischemia evaluation, nor regarding the LVESV index and the preoperative hemodynamic parameters (CI, PCWP, Papi). Without this information, it is difficult to conclude the real impact of MCS timing. Not all patients with ischemic cardiomyopathy are necessarily extremely high risk. It is likely that a preoperative evaluation that combines the anatomic, functional, morphologic, and hemodynamic areas will lead to a more precise indication for the timing and type of MCS in this complex population, thus resulting in more favorable survival.

Although randomized data are lacking, observational studies and expert consensus suggest that preoperative RV support may reduce perioperative RV failure, improve hemodynamic stability during induction and separation from bypass, and enhance postoperative recovery. Early stabilization of RV function may also expand the pool of patients who can safely undergo CABG despite advanced biventricular dysfunction [19].

The debate is ongoing; published small cohorts showed modestly favorable results with prophylactic MCS [24,25]. The rationale behind preemptive left ventricular unloading is to mitigate the effects of the cardiopulmonary bypass while waiting for the ventricle to recover, thereby maintaining end-organ perfusion and avoiding high doses of vasopressors.

Preoperative mechanical circulatory support (MCS) is increasingly employed in patients with advanced ischemic cardiomyopathy undergoing coronary artery bypass grafting (CABG) to stabilize hemodynamics and mitigate evolving end-organ dysfunction. Contemporary multicenter data demonstrate that patients requiring preoperative support—most commonly with intra-aortic balloon pump (IABP), Impella, or extracorporeal membrane oxygenation (ECMO)—present with significantly lower cardiac index and mixed venous oxygen saturation, reflecting impaired systemic perfusion and heightened risk for renal, hepatic, and metabolic compromise [26]. Importantly, planned preoperative MCS is associated with postoperative and mid-term survival comparable to unsupported high-risk patients, whereas the need for postoperative rescue MCS portends markedly worse outcomes, underscoring the protective role of early hemodynamic optimization. Recent reviews further highlight that preoperative unloading and augmentation of perfusion may reduce myocardial oxygen demand, improve metabolic reserve, and prevent irreversible organ injury prior to surgical revascularization. Collectively, these findings support a paradigm in which selective, proactive MCS deployment serves as a bridge to definitive surgical therapy in patients with advanced ischemic cardiomyopathy and marginal end-organ function [26,27].

### 2.8. Limitations of Current Mcs Evidence and Sources of Bias on Advanced Icm and CABG

Most of the available evidence on MCS in patients with ischemic cardiomyopathy undergoing CABG is observational, derived from retrospective registries, administrative databases, and small single-center series. As such, these data are inherently subject to selection bias and confounding by indication: patients selected for prophylactic or early MCS often differ systematically from those managed without support or with rescue strategies, including in hemodynamic profile, comorbidity burden, and perceived surgical risk. In studies such as those by Singh et al. and Sommer et al., the apparent survival advantage associated with early or prophylactic MCS must therefore be interpreted cautiously, as unmeasured confounders and center-specific practice patterns may influence both the decision to implant and the observed outcomes.

Furthermore, many reports present unadjusted mortality rates without comprehensive multivariable adjustment or propensity matching, limiting the ability to infer a causal effect of MCS timing or device type. Even when adjusted analyses are performed, residual confounding remains likely. Early implantation studies may also be affected by survivorship bias, as patients who live long enough and are stable enough to receive preoperative MCS may inherently have a more favorable trajectory than those who deteriorate rapidly and require emergent rescue support [28].

The distinction between prophylactic and rescue MCS is particularly important: while observational data suggest that planned preoperative support is associated with better outcomes than postoperative rescue, this may reflect differences in baseline risk and timing of decompensation rather than a pure treatment effect. Finally, there are no randomized trials specifically addressing MCS strategies in CABG for ischemic cardiomyopathy, and extrapolation from broader cardiogenic shock or PCI populations has important limitations. Collectively, these considerations underscore that current MCS data in this setting should be viewed as hypothesis-generating and support the need for prospective, rigorously designed studies to define optimal patient selection, timing, and device choice.

### 2.9. Mcs Devices

MCS devices are employed in various clinical scenarios related to CABG, specifically in ischemic cardiomyopathy. Their use is focused prophylactically or intraoperatively in patients with advanced ischemic cardiomyopathy; typically in patients presenting with clinical signs of heart failure. New York Heart Association (NYHA) class ≥III is associated with a higher need for MCS (15.9% vs. 1.9%, *p* = 0.011) [29]. Another common scenario in this advanced disease is the prophylactic use of MCS at the induction of anesthesia in patients with critical coronary anatomy and severely impaired left ventricular function. Lower preoperative EF is an independent risk factor for requiring postoperative MCS, with an odds ratio of 0.93 per unit increase in EF (*p* = 0.01) [4]. An LVEF < 0.40 is associated with a higher need for MCS (36.4% vs. 6.7%, *p* = 0.011) [22]. Finally, in advanced ischemic cardiomyopathy with marginal end-organ function, proactive MCS deployment serves to stabilize hemodynamics and mitigate evolving end-organ dysfunction. Other indications for MCS in CABG (whose detailed analysis is beyond the scope of this review) include:

- Preoperatively: acute cardiogenic shock or intractable angina with hemodynamic instability, which are primary triggers for considering preoperative or prophylactic MCS [4].
- EuroSCORE II: a EuroSCORE II >20% is associated with a higher frequency of MCS use (20.0% vs. 4.9%, *p* = 0.017) [22].
- Intraoperatively: failure to wean from cardiopulmonary bypass.
- Postoperatively: post-cardiotomy shock. Planned, early use of MCS in high-risk patients is associated with better outcomes compared with unplanned, postoperative MCS, which is linked to higher morbidity and mortality [4]. This is probably related to avoiding cardiogenic shock in the early postoperative period.

Mechanical circulatory support for advanced ischemic cardiomyopathy in CABG has undergone significant evolution, with a shift from IABP toward increased use of catheter-based devices (i.e., axial flow pumps) and ECMO. These changes reflect advances in technology, improved understanding of device-specific benefits and risks, and a focus on individualized patient care. Ongoing research and attention to disparities in device utilization are essential to optimize outcomes for high-risk CABG patients [23].

The choice of device and timing is tailored to the patient’s hemodynamic status, underlying cardiac pathology, hospital resources, and the physician’s preference. The risk-benefit profile must be carefully considered, especially in high-risk populations. There are ongoing disparities in access and utilization of MCS devices, highlighting the need for equitable application of advanced therapies [30,31].

### 2.10. Comparative Outcomes and Device-Specific Insights

#### 2.10.1. Intra-Aortic Balloon Pump (IABP)

The intra-aortic balloon pump (IABP) continues to be the most frequently used MCS device.

Mechanism: Diastolic inflation and systolic deflation provide counterpulsation.

Hemodynamic effects: Reduced LV afterload, enhanced coronary perfusion, modest CO augmentation.

Indications in CABG: Preoperative stabilization in low-output states; adjunct during separation from bypass.

Evidence: Despite declining use (74.6% to 64.6% from 2011 to 2022), IABP remains the most common device and is associated with lower operative mortality (18.0% vs. 24.4%; adjusted OR 0.76) [32].

Complications: Vascular injury, bleeding, infection, thromboembolism.

Limitations: Limited support; no direct LV unloading; observational evidence with confounding by indication. Benefits include reduced ventricular afterload, improved coronary diastolic flow, and enhanced subendocardial perfusion. However, IABP use carries risks such as aortic rupture and pulmonary embolism. There are significant sociodemographic disparities in IABP utilization, with lower use among women and patients in certain regions, and higher use among Hispanic and Asian patients [30].

#### 2.10.2. Impella

Preoperative MCS use increased from 1.3% to 11.3% during the same period. The use of catheter-based MCS, including devices such as Impella, is rising and provides hemodynamic support superior to that of the IABP. Impella has been increasingly reported as an adjunct during CABG, particularly for direct left ventricular support [23,33]. The device consists of a catheter-mounted micro-axial impeller that displaces blood from the left ventricle into the ascending aorta [33]. It delivers up to 5.5 L/min of flow, reducing pulmonary congestion and improving systemic perfusion.

Impella offers several notable advantages, including direct left ventricular unloading, reduction in pulmonary capillary wedge pressure, and decreased myocardial oxygen consumption, which may facilitate myocardial recovery. Its high antegrade flow, capacity for prolonged support, and the ability to mobilize patients with upper-extremity placement further enhance its utility. These features are particularly beneficial when used as a bridge to recovery, a bridge to decision, or a bridge to durable left ventricular assist device or heart transplantation [34]. Impella 5.5 is approved for use for up to 14 days by the Food and Drug Administration (FDA) in the United States and has the CE marking approval for use for up to 30 days in Europe.

Absolute contraindications for use of Impella 5.5 include: mechanical aortic valve (AV) prosthesis, AV endocarditis, free-floating/mobile thrombus in the LV, stent in the ascending aorta and aortic dissection. Relative contraindications include AV stenosis or calcification, severe peripheral arterial disease or lumen of <7 mm precluding its placement in the axillary artery, severe AV insufficiency or LV rupture. The most common complications are: bleeding, hemolysis, right ventricular failure, stroke, aortic valve or vascular injury, and less frequently, pump thrombosis [34].

Evidence: Catheter-based tMCS use increased from 1.3% to 11.3%. Observational studies suggest improved outcomes with proactive rather than rescue use, though limited by small samples, selection bias, and lack of viability/hemodynamic stratification. Impella 5.5 allows for prolonged, higher-flow support (FDA-approved 14 days; CE-marked 30 days).

#### 2.10.3. Extracorporeal Membrane Oxygenation (Ecmo)

ECMO is usually reserved for the highest risk cases; its use is rare in the clinical setting of advanced ischemic cardiomyopathy. ECMO is the modality of choice in post-cardiotomy shock, particularly in the presence of right ventricular failure or when other MCS devices are insufficient. Although its use remains uncommon, it is increasing, and ECMO may be preferred over right ventricular assist devices for certain post-transplant or post-CABG complications [32].

Mechanism: VA-ECMO provides full cardiopulmonary support via extracorporeal oxygenation and arterial return.

Hemodynamic Effects: Supports both ventricles; increases systemic perfusion; increases LV afterload unless vented.

Indications in CABG: Rare preoperatively; primarily for postcardiotomy shock or severe biventricular failure.

Evidence: Survival after postcardiotomy VA-ECMO ranges from 25 to 42%. CABG-specific data are limited and derived from mixed shock cohorts.

Complications: Major bleeding, limb ischemia, stroke, infection, LV distention, multiorgan failure.

Limitations: High morbidity, resource-intensive, limited role in isolated LV failure, and lack of CABG-specific evidence [35].

As we described previously, randomized data are lacking, and observational studies and expert consensus suggest that preoperative RV assessment is essential, as RV dysfunction may necessitate temporary RV support (Impella RP, dual-lumen cannula like protekduo or Spectrum Medical) to prevent perioperative RV failure and facilitate separation from bypass. Early stabilization of RV function may expand the pool of patients eligible for CABG despite advanced biventricular dysfunction [36]. Small cohorts have reported modestly favorable outcomes with prophylactic MCS, based on the rationale that preemptive LV unloading reduces myocardial oxygen demand, preserves end-organ perfusion, and mitigates the physiologic stress of cardiopulmonary bypass. Contemporary multicenter data show that patients requiring preoperative support present with lower cardiac index and mixed venous oxygen saturation, reflecting impaired systemic perfusion. Importantly, planned preoperative MCS is associated with postoperative and mid-term survival comparable to unsupported high-risk patients, whereas postoperative rescue MCS is associated with markedly worse outcomes. Collectively, these findings support a selective, physiology-guided approach to preoperative MCS as a bridge to definitive surgical revascularization in advanced ischemic cardiomyopathy.

Careful judgment regarding the indications and choices for MCS has the potential to improve the safety of CABG in high-risk patients (Figure 1).

This algorithm outlines a decision-making strategy for patients with advanced ischemic cardiomyopathy (ICM) who are candidates for coronary artery bypass grafting (CABG), focusing on identifying high-risk features and selecting appropriate mechanical circulatory support (MCS) strategies.

The first step evaluates the presence of active ischemia, defined by unstable angina or poorly tolerated ventricular arrhythmias. Patients with active ischemia are assessed for cardiogenic shock. In those with shock accompanied by elevated lactate (≥2.5 mmol/L) or evidence of organ dysfunction, escalation to advanced mechanical support is recommended, including Impella CP as a bridge to Impella 5.5/5.0 (surgical support) or consideration of veno-arterial extracorporeal membrane oxygenation (VA-ECMO). In the absence of severe metabolic compromise, intra-aortic balloon pump (IABP) or Impella CP may be considered.

Patients without active ischemia and with a compensated hemodynamic profile are stratified according to left ventricular structural and functional parameters, including LVEF ≤ 25%, LVEDD > 6.5 cm, LVEDV index > 120 mL/m^2^, LVESV ≥ 100 mL, LVESV index > 70 mL/m^2^, or severe mitral regurgitation, identifying individuals at high operative risk. These patients are further evaluated for pulmonary congestion (PCP ≥ 18 mmHg) and right ventricular function.

Markers of right ventricular dysfunction include PAPI < 1.5–2.0, moderate-to-severe pulmonary hypertension, TAPSE ≤ 12 mm, RVFAC < 25%, RV free wall strain ≥ 15%, RVEF (MRI) < 30%, CVP ≥ 15 mmHg, or severe tricuspid regurgitation. When present, medical optimization is initially pursued. If there is inadequate response, right ventricular assist device (RVAD) strategies should be considered, including dual-lumen cannulas (e.g., Spectrum Medical or ProtekDuo Plus) or Impella surgical support.

In patients without significant right ventricular dysfunction or who respond adequately to medical optimization, Impella 5.5 (Impella 5.5) may be used as a prophylactic or perioperative mechanical support strategy to facilitate safe CABG in this high-risk population. Well-designed, long-term, prospective studies are needed to evaluate its impact on patient outcomes. Temporis filia Veritas (“Truth as the daughter of time”), an ancient proverb, reflects the idea that truth often reveals itself only with the passage of time.

## 3. Conclusions

Mechanical circulatory support has evolved from a rescue intervention to a planned strategy that expands the physiological reserve of patients with advanced ischemic cardiomyopathy undergoing coronary artery bypass grafting. By stabilizing hemodynamics, preserving end-organ perfusion, and unloading the failing ventricle, MCS creates conditions in which surgical revascularization can meaningfully influence postoperative recovery. Left ventricular ejection fraction and heart failure severity remain the most consistent preoperative predictors of perioperative support requirements.

Contemporary imaging plays a central role in patient selection. PET-FDG and CMR-LGE provide high sensitivity for detecting viable myocardium and quantifying scar transmurality, while dobutamine stress echocardiography offers high specificity for contractile reserve. SPECT perfusion imaging remains widely used for assessing ischemic burden. Across modalities, segments with preserved metabolism, limited scar (<50% transmurality), or demonstrable contractile reserve show the greatest likelihood of functional recovery after revascularization. These imaging-derived parameters—viability, ischemia, and scar burden—have become integral to identifying CABG candidates most likely to benefit from surgical revascularization.

Evidence consistently shows that anticipatory and planned support strategies outperform reactive rescue approaches, reinforcing the importance of early identification of patients at risk for hemodynamic instability. Device selection, however, remains variable across institutions, and long-term outcomes associated with perioperative support require further study.

Within this context, Impella 5.5 has shown particular benefit in patients with severely reduced LVEF, large ischemic burden, advanced LV ischemic remodulation or anticipated difficulty separating from cardiopulmonary bypass, providing higher flow and more effective ventricular unloading than earlier-generation devices. Pulmonary artery pressure measurements further refine risk assessment, with elevated PAP strongly associated with the need for perioperative support. Right ventricular evaluation (with several parameters and indexed by echo) is equally important, as RV dysfunction—whether due to pulmonary hypertension, ischemia, or chronic pressure overload—may necessitate temporary RV support using devices such as Impella RP or dual-lumen cannulas.

As surgical candidates become older and more complex, integrating physiologic, imaging, and hemodynamic data into standardized algorithms will be essential. The field continues to evolve, with a shift from intra-aortic balloon pump reliance toward broader adoption of devices such as the Impella 5.5. Ongoing research, improved comparative data, and attention to disparities in device utilization will be critical to optimizing outcomes in high-risk CABG.

When deployed with discernment, these devices extend the space in which recovery becomes possible, transforming a precarious operation into a viable path toward renewed function. Continued innovation, rigorous inquiry, and multidisciplinary collaboration will be essential to ensure the best surgical results in patients with advanced ischemic cardiomyopathy.

## Figures and Tables

**Table 1:** Comparative table of imaging modalities for viability, ischemia, and scar and Sensitivity and specificity values.

Modality	Sensitivity	Specifity	Myocardial Viability	Myocardial Ischemia	Myocardial Scar
**FDG-PET**	~90–92	~60–65	High accuracy (metabolic gold standard). Detects hibernating myocardium with sensitivity ~85–90%.	Moderate. Best when combined with perfusion (perfusion–metabolism mismatch).	Low–moderate. Does not characterize fibrosis; it identifies absent metabolism.
**SPECT (Tc-99mm Thallium-201)**	~85–88	~50–55	Moderate sensitivity (70–80%). Lower specificity than PET.	Good for inducible ischemia; widely available.	Limited for small or patchy scar; lower spatial resolution.
**CMR with LGE**	~90–95	~45–55	Defines viability based on scar transmurally; excellent correlation with functional recovery.	Moderate for ischemia (requires stress perfusion).	Gold standard for scar: high resolution; distinguishes subendocardial, transmural, and patchy fibrosis.
**Stress perfusión CMR**	~85–90	~70	Indirect: identifies myocardium with preserved perfusion.	High accuracy for ischemia; comparable or superior to SPECT.	Does not detect scar without LGE
**Dobutamine stress echocardiography**	~78–82	~75–80	Good for viability in severely dysfunctional segments; sensitivity ~75–85%.	Moderate for inducible ischemia.	Does not directly detect scar; it is inferred from the absence of contractility.
**Coronary CT + perfusion**	72–82	75–86	Limited for viability; emerging.	Good for ischemia when combined with dynamic perfusion.	Detects scar with advanced techniques (late iodine enhancement), but it is inferior to CMR.

Viability: Most accurate: *FDG-PET* (metabolism) and *CMR with LGE* (scar transmurality), PET identifies metabolic hibernation; CMR predicts recovery based on scar extent. Ischemia: Most accurate: *Stress perfusion CMR* and *PET perfusion*; SPECT remains the global standard due to availability. Scar: gold standard: Cmr with Lge. Provides detailed characterization of ischemic vs. non-ischemic fibrosis.

**Table 2:** Modality, predominant population in meta-analyses and clinical relevance in CABG candidates.

Modality	Predominant Population in Meta-Analyses	Clinical Relevance in CABG Candidates
**FDG-PET perfusion/metabolism**	Ischemic LV dysfunction; PCI/CABG cohorts	Highest sensitivity for hibernating myocardium; excellent for ruling out transmural scar. Moderate specificity may overestimate viability, but valuable in high-risk CABG candidates where missing viable tissue is unacceptable.
**Thallium-201 SPECT**	Chronic ischemia; reduced LVEF; mixed revascularization	High sensitivity but low specificity; tends to overcall viability. Useful in resource-limited settings but less discriminative when surgical risk is high.
**Tc-99m SPECT (MIBI/Tetrofosmin)**	Stable ischemic cardiomyopathy; reduced LVEF	Balanced but less sensitive than PET or CMR. More specific than thallium. Helpful when integrated with wall-motion and clinical data to refine CABG decisions.
**Dobutamine stress echocardiography**	Ischemic cardiomyopathy; reduced LVEF; CABG cohorts	High specificity with moderate sensitivity. Useful for avoiding CABG in clearly non-viable territories. Limited by acoustic windows and operator dependence.
**CMR with LGE**	Chronic ischemic LV dysfunction; revascularization candidates	Excellent for quantifying scar burden and transmurally. Very high sensitivity for predicting recovery; moderate specificity because intermediate LGE may not improve. Crucial for mapping territories before CABG in severely depressed ventricles.
**CMR with dobutamine**	Advanced ischemic cardiomyopathy	Similar to dobutamine echo but with superior spatial resolution. Useful when combining contractile reserve with scar assessment in a single modality.
**Stress perfusion CMR + LGE**	Chronic ischemia; reduced LVEF; integrated evaluation	Simultaneously evaluates ischemia and scar. Particularly helpful for determining which territories merit grafting and whether complete vs limited CABG is appropriate.
**PET perfusion (NH_3_, Rb-82) ± FDG**	Multivessel disease; reduced LVEF; revascularization candidates	Adds quantification of flow and flow reserve, distinguishing hibernation from fixed microvascular dysfunction. Highly informative for high-risk CABG selection, though limited availability.

## Data Availability

No data was created for this review.

## Abbreviations

The following abbreviations are used in this manuscript:

CABG	coronary artery bypass grafting
ICM	ischemic cardiomyopathy
MCS	mechanical circulatory support
IABP	intra-aortic balloon pump
VA-ECMO	veno-arterial extracorporeal membrane oxygenation
PAPI	pulmonary artery pulsatility index
TAPSE	tricuspid annular plane systolic excursion
RVFAC	right ventricular fractional area change
CVP	central venous pressure
RVAD	right ventricular assist device
